# Total output and switching in category fluency successfully discriminates Alzheimer's disease from Mild Cognitive Impairment, but not from frontotemporal dementia

**DOI:** 10.1590/1980-57642015DN93000007

**Published:** 2015

**Authors:** Siddharth Ramanan, Jwala Narayanan, Tanya Perpetua D'Souza, Kavita Shivani Malik, Ellajosyula Ratnavalli

**Affiliations:** 1 Department of Neurology, Manipal Hospitals, Bangalore, India.; 2 Department of Clinical Neuropsychology, Annasawmy Mudaliar General Hospital.; 3 Department of Neurology, Annasawmy Mudaliar General Hospital, Bangalore, India.

**Keywords:** verbal fluency, clustering, switching, dementia, mild cognitive impairment

## Abstract

Verbal fluency tasks require generation of words beginning with a letter (phonemic fluency; PF) or from a category (category fluency; CF) within a limited time period. Generally, total output on CF has been used to discriminate Mild Cognitive Impairment (MCI) from Alzheimer's disease (AD), while poor PF has been used as a marker for behavioral-variant frontotemporal dementia (bvFTD). However, in the absence of this disparate performance, further characterization of the task becomes necessary.

**Objective:**

We examined whether fluency, as well as its components, clustering (successively generated words belonging to a category) and switching (shifting between categories) carried diagnostic utility in discriminating AD from MCI and bvFTD.

**Methods:**

PF (letter 'P') and CF ('animals') tasks were administered in English to patients with MCI (n=25), AD (n=37), and bvFTD (n=17). Clustering and switching scores were calculated using established criteria.

**Results:**

Our findings suggested that up to 85% of AD and MCI could be successfully discriminated based on total number of responses and switching in CF alone. PF-CF disparity was not noted in AD or bvFTD. Performance on clustering or switching also proved insufficient to discriminate AD from bvFTD.

**Conclusion:**

Switching was found to be useful when differentiating AD from MCI. In AD and bvFTD, the course of progression of the disease may lead to attenuation of total number of responses produced on both tasks to an extent where clustering and switching may not be useful measures to discriminate these dementias from each other.

## Abstract (pt)

Tarefas de fluência verbal requerem geração de palavras iniciadas por letras (fluência fonêmica; FF) ou por categoria (fluência por categoria; FC) dentro de um período limitado de tempo. Geralmente, a produção total na FC tem sido usada para discriminar o comprometimento cognitivo leve (CCL) da doença de Alzheimer, enquanto que, uma produção pobre em FF tem sido usada como marcador da variante comportamental da demência frontotemporal (vcDFT). Todavia, na ausência desta desproporção melhor caracterização torna-se necessária.

**Objetivo:**

Examinar se a fluência e seus componentes, agrupamentos (geração sucessiva de palavras pertencentes a uma categoria) e mudança (alternância entre categorias) teriam utilidade na discriminação entre DA, CCL e vcDFT.

**Métodos:**

Tarefas de FF (letra P) e FC (animais) foram administradas em inglês a pacientes com CCL (n=25), DA (n=37) e vcDFT (n=17). Escores de agrupamentos e alternância foram calculados através dos critérios estabelecidos.

**Resultados:**

Nossos achados sugeriram que 85% dos DA e CCL puderam ser discriminados com sucesso, baseando-se no número total de respostas e alternância na FC. A disparidade FF e FC não foi notada em DA ou vcDFT. O desempenho em agrupamento ou alternância também se provaram insuficientes para discriminar DA de vcDFT.

**Conclusão:**

Alternância foi útil na diferenciação DA de CCL. Em pacientes com DA e vcDFT a progressão das doenças podem levar à atenuação do número total de respostas produzidas em ambas as tarefas de modo que o agrupamento e alternância podem não ser medidas úteis na discriminação destas demências entre si.

## INTRODUCTION

Verbal fluency (VF) tasks require participants to generate as many responses as possible from a letter (phonemic fluency; PF) or category (category fluency; CF) within a limited time period (60 seconds). While both tasks are mediated by processing speed, verbal knowledge, and executive control,^1^ each fluency task also taps one of these functions to a greater extent. For example, PF largely relies on efficient frontal functions of self-initiation, controlled word search and retrieval, and monitoring and inhibition of responses, while CF imposes greater demands on temporal functions of integrity in semantic knowledge.^2,3^

To examine qualitative performance on VF, Troyer, Moscovitch, and Winocur^4^ devised a two-component method of clustering and switching for scoring VF responses. Clusters consist of consecutively generated words that share phonemic or semantic similarity. Clusters in PF for example, may consist of consecutively generated rhyming words (e.g. band, brand), homonyms (e.g. pair, pear), or words that share the same first two letters (e.g. postman, potter, port). Clusters in CF may consist of consecutively generated words that belong to the same semantic subcategory (e.g. *cat, dog* belong to *domestic animals*; *shark, whale* belong to *aquatic animals*). Switches involve shifting from one cluster or sub-category to another. Current literature suggests that clustering relies more on efficient temporal lobe functions of categorization, while switching taps frontal functions of shifting and cognitive flexibility.^4^ Optimal VF performance in healthy adults would thus involve exhaustive production of responses belonging to a particular cluster followed by switching to another cluster.^4^ Conversely, producing fewer CF responses and smaller clusters may suggest temporal lobe dysfunction (seen in Alzheimer's disease; AD), while a lower PF output and less frequent switches may indicate frontal lobe dysfunction (seen in behavioral-variant frontotemporal dementia; bvFTD).

Given this, while one would expect a frontal dementia such as bvFTD to display reduced PF and AD to display reduced CF responses, such sharp PF-CF discrepancies may not always be noted. In fact, recent literature^5^ has suggested that bvFTD patients may perform similarly on both VF tasks, suggesting that the pathology and the progression of the condition may affect total output on VF equally. In such cases, it becomes important to investigate whether clustering and switching can aid in accurate diagnosis of dementias and differentiate a dementia such as AD from a preclinical condition like Mild Cognitive Impairment (MCI). Investigating this aspect, previous studies have found switching to be useful,^6,7^ but their stance on clustering and its utility in discriminating AD from MCI have been equivocal. One study on CF performed in Mandarin, endorsed its use in discriminating these conditions,^6^ while another study where both VF tasks were administered in English found no utility of clustering in PF or CF for discriminating AD from MCI.^7^ Though methodological differences could explain this disparity in findings, it becomes important to not only find more evidence supporting or undermining the use of these tasks in AD vs. MCI discrimination, but also to extend the same findings to other neurodegenerative dementias. This is especially important as both clustering and switching in bvFTD, as well as their ability to discriminate bvFTD from AD, have not hitherto been studied. Presumably, if PF-CF discrepancy proves unable to discriminate an AD from a bvFTD patient, one would expect clustering and switching to be more useful in differentiating the two. The results of such findings would be especially helpful for accurate diagnosis of these conditions at a clinic, as previous classification criteria have been known to occasionally fail in discriminating FTD from AD.^8^ More importantly, it would clarify whether a quick analysis of clusters and switches could serve as a sufficiently good cognitive screen to differentiate AD from bvFTD.

The current study therefore attempted to explore whether clustering and switching could help discriminate between two dementias, as well as MCI from AD. It was hypothesized that bvFTD patients would perform poorer at switching but better on clustering and an inverse pattern should be true of AD. Secondly, given the relatively weak discriminatory value of clustering, it was hypothesized that MCI and AD can be best discriminated based solely on switching and total number of responses on VF tasks, although the diagnostic value of clustering between MCI-AD was also adequately explored.

## METHODS

**Participants.** All patients included in this study presented at the Memory Clinic, Manipal Hospitals, Bangalore with memory complaints. The control sample consisted of caregivers who accompanied patients. All our participants were multilingual non-native English speakers (speaking different languages such as Hindi, Kannada, Tamil, Telugu, Malayalam, and Bengali) but had completed their school and collegiate education in English. Despite the heterogeneity in spoken languages, all participants were fluent in English and hence this language was chosen for test administration. Ethics approval was obtained from the Hospital Ethics Committee and written informed consent was obtained from all patients and/or their caregivers. Participants with previous history of epilepsy and major psychiatric disorders were excluded. Addenbrooke's Cognitive Examination – III (ACE – III)^9^ was used as a cognitive screen and the Clinical Dementia Rating (CDR)^10^ scale was used to grade dementia severity in all participants. Sample characteristics of participants are reported in Table 1.

**Table 1:** Sample characteristics.

	Controls Mean (SD)	MCI Mean (SD)	AD Mean (SD)	bvFTD Mean (SD)	Group effect
N	35	25	37	17	
Age	67.74 (7.39)	69.36 (6.79)	72.43 (8.06)	67.00 (11.51)	n.s.
Education, years	14.22 (2.53)	14.20 (2.08)	14.88 (2.29)	15.76 (1.56)	n.s.
Disease duration, months	–	18.41^b^,^c^ (18.18)	34.55^a^ (30.67)	36.31^a^ (20.89)	*
ACE score	93.77 (5.04)	83.60**^b^,^c^ (9.28)	62.08***^a^ (13.31)	62.28***^a^ (19.57)	***
Clinical Dementia Rating	0.00 (0.00)	0.41***^b^,^c^ (0.28)	1.08***^a^,^c^ (0.47)	1.37***^a^,^b^ (0.69)	***

Asterisks indicate significant differences in relation to the control group. Letters in superscript indicate significant differences between groups on post hoc testing (using false discovery rate).

a Significantly different from MCI;

b Significantly different from AD;

c Significantly different from FTD;

* p<0.05;

** p<0.01;

*** p<0.001; ns: not significant. ACE: Addenbrooke’s Cognitive Examination – III.

The total sample size consisted of 114 participants. Thirty-five neurologically intact participants served as the control group. Twenty-five patients were classified as MCI as per Petersen's criteria.^11^ Thirty-seven patients met the criteria for probable AD as per the International revised consensus criteria.^12^ Seventeen patients were classified as bvFTD as per the revised criteria.^13^

**Methods and scoring procedure.** The letter 'P' and the category 'animals' were used to assess PF and CF, respectively. Participants were asked to name as many words as possible in English starting with the letter 'P' (excluding names of people, places, or suffixes for the same word – e.g. *play, playing, player*) or belonging to the category 'animals' in 60 seconds. Responses were recorded verbatim.

Responses were scored in a manner similar to that employed in Troyer et al.^4^ We therefore obtained three scores on each of the fluency tests:

[A] total number of words generated, excluding errors and repetitions;
[B] mean cluster size; and
[C] number of switches.

As mentioned earlier, clusters on PF tasks consisted of consecutively generated rhyming words, homonyms, words that shared the same first two letters, or the same first and last sounds. In CF, clusters consisted of consecutively generated words that belonged to the same semantic subcategory. Cluster size was counted from the second response in each cluster. Mean cluster size was derived for each fluency task. Switches were calculated as number of transitions between clusters that included single words. Errors and repetitions were excluded from total word output for each fluency task, but were included in calculations of cluster size. It should be noted that semantic subcategories defined in our study differed from those of previous studies due to cultural differences (see Appendix).

Two independent raters scored all clustering and switching performance scores separately. Inter-rater reliability was calculated using Intraclass Correlation Coefficients and correlations for mean cluster size and number of switches for both VF tasks between both raters exceeded 0.90.

**Statistical analyses.** Results were analyzed using R Studio v2.13.1. Following descriptive statistics, an analysis of variance (ANOVA) was employed to determine overall differences between groups for demographic variables, whereas an analysis of covariance (ANCOVA) was employed to determine mean differences between groups for total number of responses, clustering and switching in both fluency measures while controlling for disease severity (CDR). Post hoc pair-wise comparisons for demographics were conducted using the 'false discovery rate' method and post hoc comparisons for VF measures were conducted using Tukey's HSD while controlling for disease severity. Effect sizes are indicated using partial eta-square (η^2^_p_).

## RESULTS

There were no differences in age or education (both p values <0.1) between groups. There was a significant effect of duration of disease [F (2, 73)=3.56; p<0.05; η^2^_p_ =0.08]. Both AD and bvFTD groups reported significantly longer duration of disease than the MCI group (both p values <0.05) but importantly, the AD and bvFTD groups had comparable disease duration (p<0.1). There was a significant difference between groups on overall ACE-III score [F (3, 102)=53.62; p<0.001; η^2^_p_=0.561] and global CDR [F (3, 108)=62.93; p<0.001; η^2^_p_ =0.63]. On the ACE-III, the controls performed better than both MCI (p<0.05) and dementia groups (p<0.001) while the MCI performed better than the AD and bvFTD groups (both p values <0.001). The AD and bvFTD groups performed comparably on the ACE (p<0.1). Within the dementia group, the bvFTD group had a significantly higher CDR than the AD group, who in turn had a higher CDR than the MCI group (both p values <0.001).

Results for performance on VF tasks are given in Table 2. An ANCOVA on VF scores indicated significant differences between groups for total number of words generated in CF [F (3, 107)=7.29; p<0.001, η^2^_p_ =0.16] but not in PF [F (3, 107)=1.19; p<0.1; η^2^_p_ =0.03] after controlling for disease severity. On total word output for the CF task, the AD group performed poorer than the MCI group, (p<0.001) but comparably to the FTD group (p<0.1). No post hoc differences were noted between groups for total number of responses generated in PF. Paired t-tests within each group indicated no significant differences (p<0.1) between PF and CF performance in the control, MCI, AD, and bvFTD groups.

**Table 2:** Performance on total word output, clustering and switching across different groups.

		Controls Mean (SD)	MCI Mean (SD)	AD Mean (SD)	bvFTD Mean (SD)	Group effect
Phonemic fluency	Number of responses	13.68 (4.80)	10.76 (4.31)	6.56 (4.73)	6.23 (6.52)	n.s.
	Switches	9.02 (4.23)	7.44 (4.04)	4.94 (3.58)	4.64 (4.68)	n.s.
	Cluster sizes	0.70 (0.89)	0.50 (0.52)	0.16 (0.23)	0.19 (0.21)	n.s.
Category fluency	Number of responses	14.45 (3.46)	11.60^b^ (3.00)	5.86***^a^ (3.57)	4.94** (4.54)	***
	Switches	5.88 (2.95)	5.32^b^ (1.97)	2.35*^a^ (2.33)	2.41 (2.34)	*
	Cluster sizes	1.48 (1.47)	0.99 (0.67)	0.81 (0.81)	0.68 (0.73)	n.s.

Asterisks indicate significant differences in relation to the control group. Letters in superscript indicate significant differences between groups (using Tukey’s HSD).

a Significantly different from MCI;

b Significantly different from AD;

* p<0.05;

** p<0.01;

*** p<0.001.

An ANCOVA examining mean cluster sizes indicated no significant group or post hoc differences in PF or CF for any of the groups (p<0.1). Mean cluster size for both PF (r=0.21, p<0.05) and CF (r=0.35, p<0.001) had low correlations with total number of PF and CF responses generated. Paired t-tests for mean cluster sizes indicated that controls [t(34)= –2.58; p<0.05], MCI [t(24)= –2.71; p<0.05], AD [t(36)= –4.96; p<0.001] and bvFTD [t(16)= –3.31; p<0.01] groups produced larger clusters in CF than PF.

ANCOVAs examining switching performance indicated group differences in CF [F (3, 107)=3.43; p<0.05, η^2^_p_ =0.08] but not PF [F (3, 107)=0.29; p<0.1, η^2^_p_ =0.008]. Post hoc differences revealed that the AD group made fewer switches than both control and MCI groups (both p values <0.05). Paired t-tests indicated that control [t(34)=4.51; p<0.001], MCI [t(24)=2.38, p<0.05], AD [t(36)=4.78; p<0.001], and bvFTD [t(16)=2.51; p<0.05] groups made more switches in PF than CF. Switching performance in both fluency measures correlated highly with total number of PF (r=0.91; p<0.001) and CF (r=0.77; p<0.001) responses. No other significant differences were documented.

**Figure 1:**
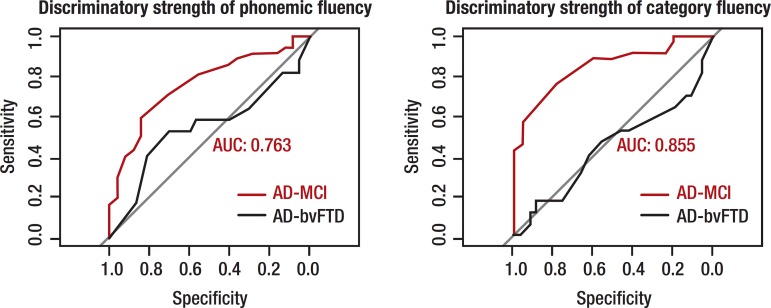
Discriminatory strength of total output on phonemic and category fluency tasks. AUC: area under the curve. Red curve indicates AD-MCI comparison, black curve indicates AD-bvFTD comparison. AUC displayed for most-sensitive group difference, in both cases, AD vs. MCI.

**Figure 2:**
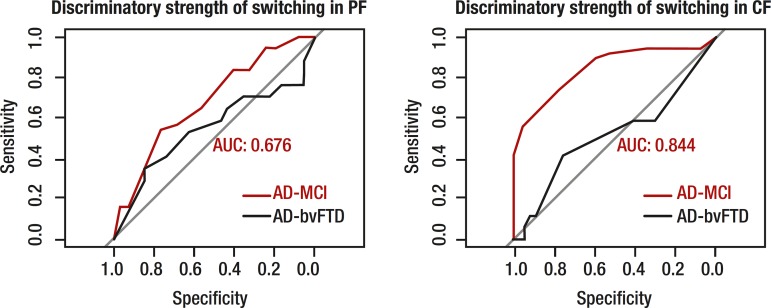
Discriminatory strength of switching on phonemic and category fluency tasks. PF: phonemic fluency; CF: category fluency; AUC: area under the curve. Red curve indicates AD-MCI comparison, black curve indicates AD-bvFTD comparison. AUC displayed for most-sensitive group difference, in both cases, AD vs. MCI.

**Logistic regression.** Logistic regression analyses were conducted to ascertain which VF measure could best discriminate different patient groups. Receiver Operating Characteristic curves for total output and switching in PF and CF are displayed in Figures 1 and 2. The two strongest discriminators between MCI and AD groups were the number of responses produced in CF and number of switches made within CF, successfully distinguishing 85.5% and 84.5% of MCI and AD patients, respectively. Between control and MCI groups, the two strongest discriminators were total number of words in CF (75%) and PF (69.5%), with switching in CF discriminating weakly (45%). Between AD and bvFTD patients, total number of responses, clustering and switching measures in PF and CF discriminated both groups within a range of 45%–54%.

## DISCUSSION

The current study attempted to determine whether clustering and switching in VF tasks could possibly serve as reliable proxies aiding discrimination of AD from MCI and from bvFTD. To this end, the scoring method suggested by Troyer et al.^4^ was adapted and our analysis was controlled for disease severity (global CDR). From our results, several important findings emerged that are discussed below.

First, our results suggest that, along with total number of CF responses generated, the number of switches within CF successfully discriminated MCI from AD to a high degree (close to 85%). Our findings replicate previous results^7^ while suggesting that these measures are also highly efficient for differential diagnosis of MCI and AD at a clinic. One earlier study found switching as well as cluster size to discriminate MCI from AD in a Mandarin-speaking patient group attempting 'supermarket' fluency as their CF task.^6^ However, the mean cluster sizes in their MCI and AD groups were much higher than those traditionally reported in other studies, which could be attributed to differences in language and choice of VF category. In contrast, our findings on the category 'animals' (commonly used by most clinics worldwide) attempted in English suggest that in the current AD sample, adequate clustering (in relation to MCI and bvFTD groups) but reduced switching in CF may be highly suggestive of a difficulty in distinguishing different semantic subcategories, rather than producing enough exemplars within each subcategory. Importantly, our findings reiterate that clustering in both VF tasks has poor diagnostic utility in discriminating MCI from AD and bvFTD from AD, while switching in CF proves to be a stronger measure, especially for discriminating MCI from AD. Combined with findings from Zhao et al.^6^ where VF was administered in Mandarin, we suggest that switching and total number of responses in CF across two of the most widely spoken languages may be useful indicators to aid clinicians in distinguishing MCI from AD.

Second, we did not note any superior or preferential performance on PF or CF tasks among any of our patient groups. This finding was contrary to earlier reports of disparately poor performance on PF by bvFTD patients and on CF by AD patients.^14^ Further investigating bvFTD and AD, we also found none of the VF measures to have superior diagnostic utility in discriminating these groups. This was surprising and contrary to our hypothesis, as we had supposed switching and clustering to be 'frontal' and 'temporal' tasks, respectively, and that the performance on these would help discriminate bvFTD from AD. There are several possible reasons for these patterns of findings. First, our bvFTD and AD groups were equally impaired when brought to the clinic, as evidenced by their comparable disease duration and ACE-III performance. This could have possibly caused global attenuation in the number of responses produced, affecting both VF tasks similarly and thereby, giving no opportunity for preferential performance on one VF task over another. Producing fewer responses would obviously give little opportunity to switch efficiently between clusters, possibly explaining comparable performance between our AD and bvFTD groups on all VF measures. In cases where participants produce few responses, clustering and switching may not be beneficial measures to discriminate groups. In such cases, it may be worth examining the time taken to produce consecutive responses within and between clusters^15^ that may inform better on strategic search and retrieval, slowed speed of processing as well as integrity of semantic knowledge in both conditions. Exploring such alternate measures are especially important, as some previous studies have acknowledged difficulty in accurately classifying some bvFTD patients who, symptom-wise, may fulfill AD criteria.^8^ Such misclassification would obviously affect the prognosis and treatment of the disease.

Noticing the overall pattern of responses, one may also argue that the mean total output in our participants may have been low because all of the participants were non-native speakers of English performing a VF task in English. Previous research by our group on healthy adults however, has indicated that regardless of self-rated proficiency in English (as L1, L2, or L3), participants performing VF in English generated a greater number of responses as compared to other Indian languages, even when they rated themselves as less fluent in English.^16^ Proficiency in English therefore, may not have been a barrier to producing more responses on VF tasks. However, this factor has yet to be explored in patients with neurodegenerative dementias.

In summary, our findings suggest that switching and total number of words generated in CF discriminate MCI and AD to a high degree. This finding has clear implications for clinicians, allowing them to differentiate MCI from AD in the clinic without formal neuropsychological testing. In such cases, the relative brevity of the CF task and ease of computing switching scores further aids clinicians, with VF tests taking no more than two minutes in total. The results from this analysis can be supplemented with presenting complaints and degree of functional impairment to arrive at a preliminary diagnosis of MCI or AD. At the same time, our findings suggest that these measures may not be useful as diagnostic cognitive markers to discriminate AD from bvFTD, however, this warrants further investigation. In these cases, short assessments of topographical memory (e.g. Four Mountains Test)^17^, social cognition,^18^ and emotion processing^19^ have been shown to be individually useful in discriminating AD from bvFTD and a combination of these may be more robust for differentiating these conditions. This issue remains to be examined further.

Despite the clinical relevance of these findings, limitations of the study include a small bvFTD sample. We also did not divide our MCI group into single and multi-domain, and amnestic and non-amnestic MCI. Similarly, we arrived at our results based on performance on one letter and one category only, whereas using multiple letters and categories may offer more stable results. Future studies should also control for different cognitive and behavioural characteristics such as apathy in bvFTD, which would further help elucidate other influences on task performance.

## APPENDIX

**Table:**

**Phonemic Fluency** **(Letter P)**	• Clusters on this task consisted of consecutively generated words sharing any of the characteristics mentioned below. The same rule was applied to responses generated in Indian languages with the phoneme /p/. • Same *first two letters*: words that shared the same first two letters, like *pay* and *pale* • Same *first and last sounds:* words that differed by the sound of a vowel, irrespective of their spelling, such as *pat*, *pet, pot* and *pit* • *Homonyms:* Similar sounding words with different spellings, such as *patients* and *patience*, as indicated by our participants • *Rhyming words:* such as *plane* and *pain*
**Category Fluency** **(Animals)**	• Clusters on this task consisted of consecutively generated words belonging to the same semantic subcategory. The subcategories defined here are relatively simple and differ from those described in earlier papers due to educational and cultural differences. The list of commonly generated examples by our participants are mentioned below: • *Aquatic animals:* dolphin, fish • *Birds:* cock, crow, dove, duck, eagle, goose, hen, kite, koel, nightingale, owl, parrot, peacock, penguin, sparrow • *Domestic animals:* ass, buffalo, bull, camel, cat, cow, dog, donkey, goat, horse, mule, rabbit, ox, pig, sheep • *Insects:* ant, bee, butterfly, cockroach • *Reptiles:* alligator, anaconda, chameleon, crocodile, cobra, frog, lizard, python, snake, tortoise, turtle, viper • *Rodents:* rat, mouse, squirrel • *Wild animals:* antelope, baboon, bear, bison, cheetah, chimpanzee, deer, elephant, fox, giraffe, gorilla, hippopotamus, hyena, jackal, kangaroo, leopard, lion, monkey, orangutan, panther, polar bear, rhinoceros, tiger, wolf, zebra
